# Recent Advances in Implantation-Based Genetic Modeling of Biliary Carcinogenesis in Mice

**DOI:** 10.3390/cancers13102292

**Published:** 2021-05-11

**Authors:** Masashi Izumiya, Shingo Kato, Yoshitaka Hippo

**Affiliations:** 1 Department of Gastroenterology, The University of Tokyo Hospital, Tokyo 113-8655, Japan; izumiya@m.u-tokyo.ac.jp; 2 Department of Clinical Cancer Genomics, Yokohama City University Hospital, Kanagawa 236-0004, Japan; shin800m@yokohama-cu.ac.jp; 3 Department of Molecular Carcinogenesis, Chiba Cancer Center Research Institute, Chiba 260-8717, Japan

**Keywords:** genetically engineered mouse, biliary tract cancer, organoid, orthotopic model, nude mouse, syngeneic, hydrodynamic injection, implantation

## Abstract

**Simple Summary:**

Biliary tract cancer (BTC) is often refractory to conventional therapeutics and is difficult to diagnose in the early stages. In addition, the pathogenesis of BTC is not fully understood, despite recent advances in cancer genome analysis. To address these issues, the development of fine disease models is critical for BTC. Although still limited in number, there are various platforms for genetic models of BTC owing to newly emerging technology. Among these, implantation-based models have recently drawn attention for their convenience, flexibility, and scalability. To highlight the relevance of this approach, we comprehensively summarize the advantages and disadvantages of BTC models developed using diverse approaches. Currently available research data on intra- and extrahepatic cholangiocarcinoma and gallbladder carcinoma are presented in this review. This information will likely help in selecting the optimal models for various applications and develop novel innovative models based on these technologies.

**Abstract:**

Epithelial cells in the biliary system can develop refractory types of cancers, which are often associated with inflammation caused by viruses, parasites, stones, and chemicals. Genomic studies have revealed recurrent genetic changes and deregulated signaling pathways in biliary tract cancer (BTC). The causal roles have been at least partly clarified using various genetically engineered mice. Technical advances in Cre-LoxP technology, together with hydrodynamic tail injection, CRISPR/Cas9 technology, in vivo electroporation, and organoid culture have enabled more precise modeling of BTC. Organoid-based genetic modeling, combined with implantation in mice, has recently drawn attention as a means to accelerate the development of BTC models. Although each model may not perfectly mimic the disease, they can complement one another, or two different approaches can be integrated to establish a novel model. In addition, a comparison of the outcomes among these models with the same genotype provides mechanistic insights into the interplay between genetic alterations and the microenvironment in the pathogenesis of BTCs. Here, we review the current status of genetic models of BTCs in mice to provide information that facilitates the wise selection of models and to inform the future development of ideal disease models.

## 1. Introduction

**Figure 1:**
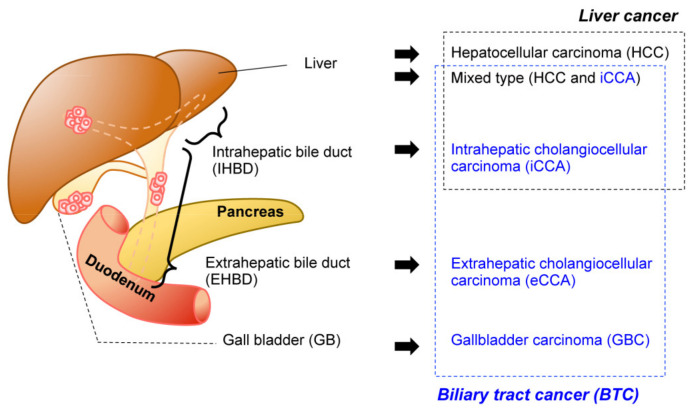
Malignancy of the hepatobiliary system. Anatomical classification of the biliary system (left). The corresponding tumors are listed (right). Biliary tract cancers (BTC) (blue) and liver cancer (black) are not necessarily mutually exclusive.

The biliary system is a network of bile ducts that collect bile produced and secreted by hepatocytes in the liver. The bile ducts merge into the common bile duct (CBD), transporting bile to the duodenum, where it aids the absorption of dietary lipids in the intestine. The gall bladder (GB) is located in the middle of this network. It functions to temporally store bile during the fast state, which in turn is secreted by GB contraction upon food intake. Biliary tract cancer (BTC) is a malignant tumorous cancer that arises from epithelial cells that cover the lumen of the bile duct. It is typically divided into three subtypes (Figure 1) based on anatomical site: intrahepatic cholangiocarcinoma (iCCA), extrahepatic cholangiocarcinoma (eCCA), and gallbladder carcinoma (GBC). While the histological diagnosis for most BTCs is adenocarcinoma, a subset of liver cancer is a biphasic tumor comprising both iCCA and hepatocellular carcinoma (HCC), thereby diagnosed as mixed type [1].

The major risk factors for BTC include chronic infection with liver fluke and primary sclerosing cholangitis. Other risk factors include chronic liver disease, stones, fibrocystic polycystic disease, chemicals, obesity, aging, and some genetic diseases, suggesting that chronic inflammation in the local biliary tract may be implicated in its pathogenesis [2]. Worldwide, iCCA cases are increasing, and eCCA cases are decreasing. The reasons for this trend remain largely unknown [3,4]. BTC is one of the most devastating cancer types, with a 5-year survival rate of approximately 10% to 30% for all patients and 2% to 3% for patients with metastatic disease. Although biliary intraepithelial neoplasia (BilIN) [5,6] is regarded as a putative pre-cancerous lesion for BTC, it can only be detected by histological examination of the tumors. Non-invasive diagnostic modalities, such as serum biomarkers or imaging, have not been developed. For early stage tumors, surgical resection followed by adjuvant chemotherapy is the first-line therapy to achieve a complete cure. In contrast, patients with advanced or metastatic disease are treated with systemic chemotherapy, radiation therapy, and local therapy, which may lead to palliative care.

Recent genomic analyses have revealed that mutations in tumor protein 53 (TP53), BReast CAncer gene (BRCA), phosphatidylinositol-4,5-bisphosphate 3-kinase catalytic subunit alpha (PIK3CA), and KRAS are commonly found in all types of BTC. In contrast, fibroblast growth factor receptor 2 (FGFR2) fusion genes and isocitrate dehydrogenase 1 (IDH1) mutations are preferentially detected in iCCA, protein kinase CAMP-activated catalytic subunit alpha (PRKACA)- and PRKACB-fusion genes in eCCA, and mutations in epidermal growth factor receptor (EGFR), ERBB3, and phosphatase and tensin homolog (PTEN) in GBC [7,8,9,10,11,12,13,14]. Hence, molecular targeted therapies with inhibitors of IDH or FGFR can be considered for patients with iCCA. Radiation therapy and immunotherapy with immune checkpoint inhibitors or chimeric antigen receptor T cell therapy may also be applied, although their efficacy remains elusive. Mouse models, particularly genetically engineered mice (GEM), have been fundamentally important in elucidating the mechanisms underlying BTC development and are a potentially powerful tool for preclinical studies.

Given the recent progress in cancer genome projects and their application to genomic medicine in clinical practice, this work comprehensively reviews the recent progress in mouse BTC models, emphasizing those that recapitulate the whole processes of BTC development by genetic approaches. Although perihilar cholangiocarcinoma (pCCA) is a distinct entity of high clinical relevance, it is not distinguished from eCCA in most genomic studies, and no mouse model has been developed for pCCA. Therefore, we adopted this classification of BTC in this review and excluded hepatocellular carcinoma (HCC) models unless the induced tumors were mixed with iCCA. Similarly, other BTC models [15,16,17] with patient-derived xenografts in immunodeficient mice, or in vivo mouse models based on the liver damage caused by chemicals or bile duct ligation were also excluded. The aim of this review is to provide a reference guide for researchers to decide which model or technology to use in BTC studies. In particular, newly emerging implantation-based models using organoids have been prioritized, recognizing their future potential in BTC modeling.

## 2. Technical Overview of Genetic Mouse Models of BTC

**Figure 2:**
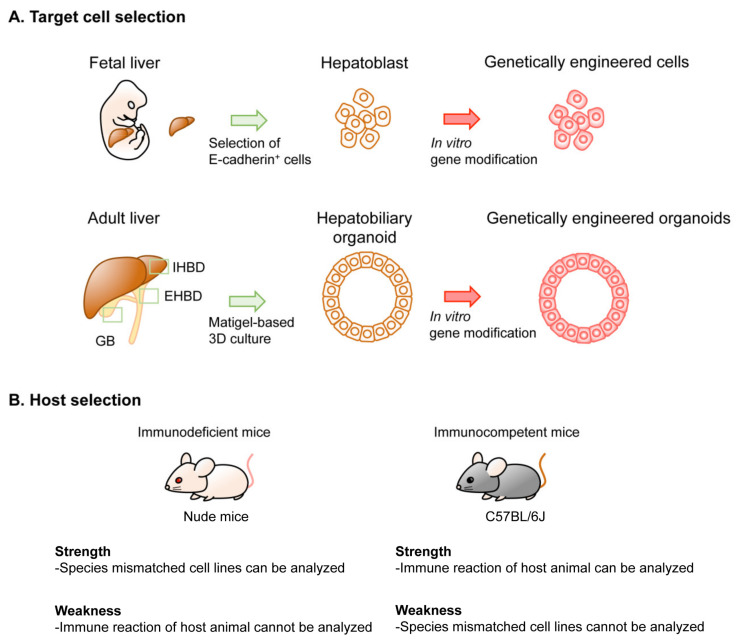
Options in implantation-based modeling of BTC. (See Table 1 for the details of each study.) As examples of options for modeling BTC, target cell selection (**A**) and host selection (**B**) are illustrated. Abbreviations are: IHBD, intrahepatic bile duct and EHBD, extrahepatic bile duct. Nude mice and C57BL/6J strain mice were used as representatives for immunodeficient mice and immunocompetent mice, respectively.

There are several different platforms for genetic modeling of BTC in mice, generating considerable variations in the latency and penetrance of tumor development, cost, equipment, technique required for the models, and the experimental settings. Thus, thorough considerations are advisable before selecting the type of model to be generated. Several options can be selected for the genetic modeling of BTC in mice. These include the target cell type, the method used in genetic engineering, the type of host mouse, and the location of tumor development (Figure 2). In this section, we review the technical aspects of these options.

### 2.1. Target Cell Type

To establish a carcinogenesis model, it is vital that pro-tumorigenic genetic aberrations are properly reconstituted in the right cell of origin of the cancer. However, the cell-of-origin of BTC has only been partially characterized, owing to the limited number of tissue- or cell lineage-specific Cre mice. Conversely, direct isolation of the organs and primary organoid culture has enabled the enrichment of organ-specific cell types, at least for biliary epithelial cells.

#### 2.1.1. Specification by Cre Mice

The Cre-LoxP system has been widely used to identify tumor-initiating cells and develop tumors in a tissue- or organ-specific manner [25,26,27,28]. In mice with conditional alleles that contain two LoxP sequences, flanked lesions were spliced upon the induced expression of Cre recombinase. Various types of transgenic or knock-in mice have been developed to date, in which Cre expression is regulated by the promoters of genes whose transcription is active only in certain types of cells or organs. The promoters that drive Cre in mouse models of BTC include, but are not limited to, *albumin*, *alpha-fetoprotein*, *cytokeratin 19* (*Krt19*), *hepatocyte nuclear factor 1 homeobox B* (*Hnf1b*), *SRY-box transcription factor 9* (*Sox9*), and *osteopontin* (*Spp1*) [29]. Local injection of adenovirus Cre or intraperitoneal injection of *β*-naphthoflavone to induce expression of Cre in *Aryl hydrocarbon receptor (Ah)*-Cre mice can also achieve genetic recombination in the conditional GEM to develop BTC [30,31,32]. However, the precise specification of epithelial cells or cell lineages remains a technical challenge.

Accumulating evidence suggests that the biliary system comprises a highly heterogeneous cell population. For example, *Sox9* is expressed throughout the bile duct, whereas *Krt19* is expressed in some of the bile ducts and pancreatic ducts [33,34]. In addition, *Alb* is expressed in bi-potential progenitors for both hepatocytes and cholangiocytes, which might account for the frequent development of iCCA mixed with HCC [35]. Moreover, it was recently reported that *Alb*-Cre achieved gene recombination in both hepatocytes and hepatic stellate cells [36]. There is also the possibility that iCCA can arise from differentiated hepatocytes that undergo transdifferentiation into cholangiocytes, as evidenced by single cell analysis of the entire liver [37,38]. eCCA may develop from the peribiliary glands of the CBD [22]. Thus, the cell-of-origin of BTC needs to be further investigated. Future development of fine-tuned specific Cre mice may pave the way for more detailed analyses to clarify the diversity of the BTC cell-of-origin.

#### 2.1.2. Specification by Physical Isolation Followed by Primary Culture

Many organs isolated from wild-type (WT) mice or conditional GEM are amenable to primary cell culture. Fetal liver cells can be maintained in vitro for a certain period of time by co-culture with irradiated fibroblasts as feeder cells. In contrast, with Matrigel-based three-dimensional (3D) culture, organoids from the adult liver and GB can be propagated almost infinitely in serum-free media supplemented with various niche factors for stem cells. These factors include EGF, noggin, and Jagged-1 [19]. Propagated organoids quickly become free of non-epithelial cells and comprise only epithelial cells from the target organs. Tissue specificity is thus readily achieved by physical separation, which reduces the need for highly specific Cre mice. However, under standard culture conditions, liver-derived organoids exclusively differentiate into cholangiocytes, unlike in vivo situations, although bi-potential progenitor cells are likely present at the beginning of organoid culture [19]. A recent study described the use of a cocktail of three small molecules, CHIR-99021, blebbistatin, and forskolin, which enabled liver organoids to retain the bi-potential differentiation capacity for many passages [39]. With this technique for the physiological culture of liver cells, the future development of novel refined carcinogenesis models may be feasible.

### 2.2. Genetic Engineering

**Figure 3:**
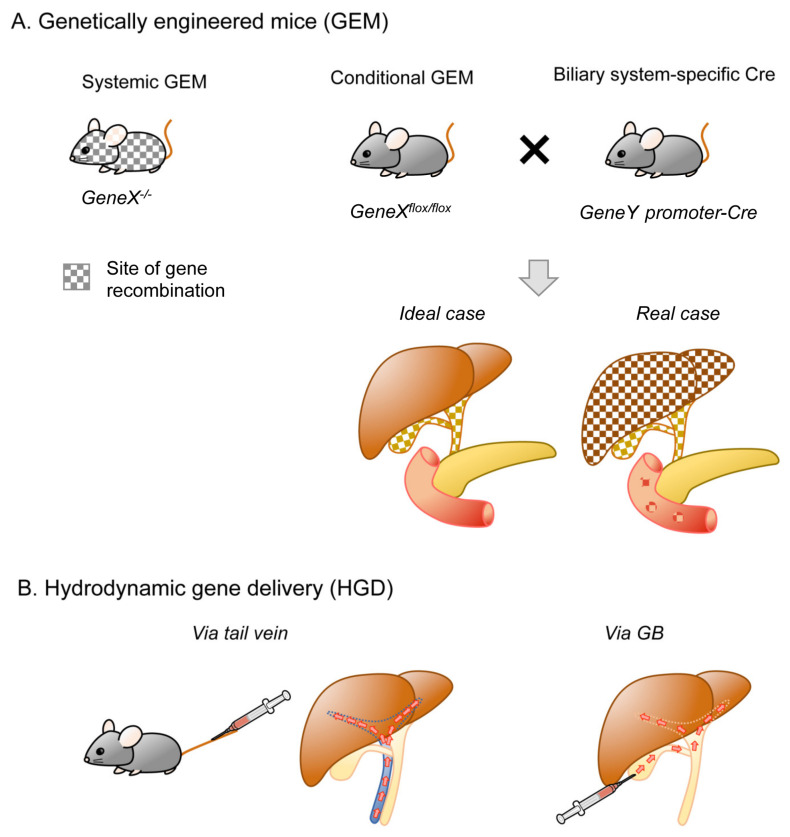
In vivo genetic engineering. (**A**). Genetically engineered mice (GEM). By crossing conditional GEM for the “gene X” and “gene Y”-Cre mice, which allow biliary system-specific recombination, resultant mice are supposed to be genetically engineered only in the biliary system (left). Note that, in reality, Cre expression could be broader (right). (**B**). Hydrodynamic gene delivery (HGD). Tail vein injection is the major means of gene delivery, while injection via GB is also reported.

Genetic engineering and gene editing can be conducted both in vivo and in vitro. However, systemic gene targeting can result in embryonic lethality or developmental anomalies in knockout mice. In addition, even if pups are born viable and healthy, tumor development in other organs may occur earlier than precede that in the organs of interest, thereby limiting the analysis of tumor development in the right target. To address these issues, gene targeting in a spatiotemporally regulated manner by applying Cre-LoxP technology to mice carrying floxed alleles has become the standard platform for GEM. More recently, CRISPR/Cas9 technology, in situ electroporation, and hydrodynamic gene delivery (HGD) have also been applied to the biliary system (Figure 3). Various combinations of these modalities have further extended the diversity of BTC modeling, although these approaches are limited.

#### 2.2.1. In Vivo Genetic Engineering

In conditional GEM, genetic engineering is already being conducted at the germline level. At a later time point, Cre recombinase excises the flanked sequence in a regulated manner in the target somatic cells with high penetrance. Conversely, hydrodynamic gene delivery and in situ electroporation can locally achieve oncogene overexpression in somatic cells with lower transduction efficiency.

##### Conditional GEM

In mice with the Cre-LoxP system, genetic recombination, such as *Alb1-Cre* in the fetal liver, is usually achieved by inducing tissue-specific expression of *Cre* during development. Alternatively, local injection of the adenovirus encoding Cre in floxed mice or tamoxifen administration in Cre-ERT2 floxed mice can achieve acute and inducible gene recombination in adults, respectively, which may be more similar to physiological situations observed in sporadic carcinogenesis in humans. Although the conditional GEM is currently the gold standard for modeling cancer, there are several caveats to be considered when establishing ideal models. First, genetic alterations that already exist across the embryonic organ might be compensated for during development at the organ level or could affect the maturation of the local immune system. In a strict sense, such GEMs may not be justified unless the aim is to model the hereditary cancer syndrome. Second, there is an issue of tissue- or cell lineage-specificity of Cre expression. Third, local induction of Cre could result in gene recombination in non-epithelial cells as well as in target epithelial cells, which is a rare situation in sporadic cancers in humans. Lastly, the generation of conditional GEM still requires labor-intensive work even with CRISPR/Cas9 technology, which significantly facilitates the development of GEM. Thus, it may be difficult to establish ideal cancer models solely by optimizing the in vivo GEM approach.

##### Hydrodynamic Gene Delivery (HGD)

HGD is a technique for the exogenous introduction of gene aberrations into hepatocytes in the liver [40,41]. By rapid injection of a high-dose nucleic acid solution through the tail vein of a mouse, the resultant high hydrostatic pressure is transmitted through the inferior vena cava and hepatic vein to the hepatocytes facing the sinusoids. Through the temporary creation of pores, expression vectors can be readily introduced into hepatocytes with efficiencies as high as 30% to 40% [41]. Together with the relatively simple procedure and anatomical features of the liver, this technique provides a unique modality for gene transfer into the liver. Although the primary target cells are hepatocytes, there is a possibility that genes are introduced into other cell types in the liver or other organs. For example, a method of injecting bile ducts into bile ducts has also been reported [42,43]. While naked DNA can be used for HGD, the *Sleeping Beauty* transposon or CRISPR/Cas9 systems are preferentially used in gene transfer for carcinogenesis research, as stable genetic traits are required during the process of tumor development.

In the BTC model using HGD, the development of liver cancers with different histologies, such as HCC, iCCA, and mixed type liver cancer, depends on the oncogenes introduced into the hepatocytes. These findings suggest that transdifferentiation of hepatocytes into biliary cells may occur or that transduction of bi-potential progenitor cells is achieved. Using the HGD approach, myristoylated (*myr-*) *AKT*, activated Notch (*NICD*), *Yap^S127A^*, dominant-negative *Fbxw7*, and *Bmi*/*Nras* were introduced in combination for the development of iCCA and mixed-type liver cancer [42,43,44,45,46,47,48,49,50,51,52,53]. WT mice are commonly used for this purpose. However, GEMs that include *Fasn^f/f^* and *p19Arf* ^−/−^ or *p19Arf* ^+/−^ have also been used to augment tumorigenicity [44,49]. It remains to be investigated whether the iCCA generated in this model is equivalent to the iCCA that originates from the normal bile duct epithelium via pre-cancerous lesions, such as BilIN. Nonetheless, it is still difficult for HGD to model eCCG and GBC, which develop outside the liver.

##### In Situ Electroporation

Gene transfer by electroporation, which is usually performed on cultured cells or fertilized eggs, is also applicable to adult tissues in vivo [54]. In introducing a gene specifically to the liver, expression vectors are injected under the liver capsule, sandwiched by the electrodes, and shocked with a pulse wave [55,56]. Using this method, gene transfer is selectively achieved in hepatocytes, but not in distal bile duct cells. Given that only iCCA develops, transdifferentiation of hepatocytes might underlie biliary carcinogenesis by electroporation. Although this technique is effective, the target cells are limited to those in the subcapsule of the peripheral liver. Interestingly, the same genetic aberrations reconstituted in the liver of *p19Arf^−/−^* mice by HGD and electroporation resulted in the development of tumors with distinct histological features, raising the possibility that the gene transfer method might critically affect the outcome [57].

#### 2.2.2. In Vitro Genetic Engineering

As the primary 2D fetal liver culture and 3D organoid culture became standard techniques, genetic engineering in these primary cells has been conducted in vitro. Compared to standard cancer cell lines, primary cells tend to be resistant to gene transduction. Therefore, some special reagents have been developed in place of polybrene, paving the way for highly efficient viral infections. Given that subsequent tumor development in mice requires a certain period of time and high transduction efficiency, genetic modifications must be stably fixed in the genome. In this regard, transfection-based knockdown with small interfering RNA (siRNA) or forced overexpression of cDNA is not common.

##### Viral Infection

Retroviruses or lentiviruses can be used to introduce cDNA encoding Cre, oncogenes, and fusion genes and short hairpin RNA (shRNA) targeting tumor suppressor genes (TSGs) into both fetal liver cells and hepatobiliary organoids. However, the simple addition of the viruses to Matrigel-embedded organoids proved insufficient for establishment of infection because Matirgel seemed to inhibit the access of these viruses to organoids [58]. In the absence of Matrigel, intact organoids were also resistant to infection, whereas dissociated cells did not survive the culture. To overcome these technical challenges, dissociated cells were seeded on solidified Matrigel for overnight co-incubation with viral particles. The cells attached to Matrigel were overlaid with Matrigel to resume 3D culture the next day. Using this approach, which integrates the culture method designated as the Matrigel bilayer organoid culture (MBOC) [59] and overnight virus infection, the transduction efficiency reached ~90% using lentivirus and ~30% using retrovirus [60]. Transduced cells can be selected with antibiotics if necessary.

As an alternative approach, the spin infection technique originally developed for non-epithelial cells [61,62,63] can be used. In this approach, dissociated cells are centrifuged with viral particles for several hours to achieve infection. In another study that used air–liquid interphase cultures, retroviral articles were directly microinjected into the lumen of organoids [64]. Expertise with this approach can influence success. By virtue of the reverse transcriptase in retrovirus and lentiviruses, transduced genes are integrated into the genome of murine cells, thereby allowing long-term assays, such as tumor development, after inoculation in other mice. The adenovirus encoding Cre can be used only for organoids with floxed alleles, as transient expression of Cre is sufficient for genetic recombination.

##### CRISPR/Cas9

The CRISPR/Cas9 system is an acquired immunity mechanism against exogenous viruses originally found in bacteria [65,66,67,68]. Being a gene editing technology with a high degree of freedom in selecting targeting sites, it has recently been applied to many fields with incremental improvements [69]. The Cas9 protein cleaves DNA by creating a blunt-ended double-strand break (DSB) in the vicinity of the protospacer-adjacent motif (PAM) and a sequence-specific guide RNA [70,71,72]. During DSB repair, which is extensively error-prone, indels are frequently inserted, resulting in a frameshift knockout of the gene [73].

However, off-target effects have been a problem with CRISPR/Cas9, where genome editing is performed on the same sequence existing outside the target site [74,75,76,77,78]. To circumvent this problem, gene editing technologies using Cas9 mutants, which can only perform single-strand breaks (SSBs), have been developed in recent years [79,80,81]. For example, the double nickase technology creates a situation similar to DSBs by inserting two single nicks in close proximity to each other. The off-target site is repaired with high accuracy because only an SSB occurs, while the target site induces a DSB, resulting in a targeted knockout [82].

In addition to conventional transfection, viral infection and electroporation are used to introduce Cas9 protein and guide RNA into cells. Although it could become complicated if single-cell cloning followed by sequencing is required, it is clear that gene knockout can be easily performed on cultured cells and has been applied to mouse and human organoids [83,84]. Regarding the CRISPR/Cas9 system, electroporation and lipofection are feasible in addition to viral infection. However, careful optimization of the protocol is generally required to avoid toxicity associated with the procedure. In addition, selection with antibiotics or inhibitors and single-cell cloning is necessary because of the low transduction efficiency and resultant heterogeneous cell population, respectively.

### 2.3. Recipient Mice

The tumorigenicity of genetically modified cells in vitro is usually determined by inoculation into other mice. As the residual immune system in the host and tumorigenicity of inoculated cells would theoretically be in a reciprocal relationship, recipient mice must be selected depending on the purpose of the study. Immunodeficient mice harboring a functional defect in the immune system to various extents have been used, particularly in the implantation of tumor xenografts and cancer cells from human patients, owing to their weak rejection capacity. Even when used for implantation of murine cells, immunodeficiency can contribute to the development of some premalignant lesions from organoids harboring weak oncogenicity, such as cysts and tiny nodules with no atypical glands [19].

Nude mice are representative immunodeficient mice, with a mutation in the *Foxn1* gene and a lack of thymus and T cells. Severe combined immunodeficiency (SCID) and non-obese diabetic (NOD)-*scid* mice with mutations in the *Prkdc* gene are more severely immunocompromised and deficient in T cells as well as B cells. NOD/Shi*-scid* IL2rγ^null^ (NOG) and NOD-*scid* gamma mice cross between NOD-*scid* mice and mice lacking the interleukin-2 (IL-2) receptor γ chain. These severely immunocompromised mice may not be necessary for mouse studies because murine cells are more prone to transformation than human cells [85,86]. In principle, upon tumor development in immunodeficient mice, the immune response of the entire host or in the local tumor area cannot be analyzed. This could become problematic, especially when the response to immune checkpoint inhibitors is the focus of this study. Collectively, nude mice may be the most suitable for mouse allograft carcinogenesis models, owing to their low cost and lack of hair, unless the focus of the study was on the immune response to tumors.

WT mice of the same inbred strain as transduced organoids are usually selected as immunocompetent recipients for implantation. Although C57BL/6J mice are commonly selected, if the GEMs are generated in other strains, multiple backcrossing with C57BL/6J will be required before the beginning of the experiments. Alternatively, the original strain for the GEM can be used, but it should be noted that the tumor predisposition varies among the strains and is organ dependent. The advantages of using syngeneic mice include the intact local and systemic immunity to tumors, the least variations in tumor development among recipients, and the applicability to evaluate the efficacy of immune checkpoint inhibitors [87]. Conversely, the tumor size and take rate of transplanted organoids could be lower than those of immunodeficient mice [88], presumably due to the anti-tumor effects of the intact T cells. Recently, humanized mice have been developed, in which the immune system is partly reconstituted by their human counterparts [89,90]. Considering the current limitation in predicting the phenotype of human cancer patients based on the responses in mouse models, humanized mice might become a useful modality, if available. However, it may still be difficult to mimic the entire human immune system given the complex network within each individual.

### 2.4. Location of Tumor Development

**Figure 4:**
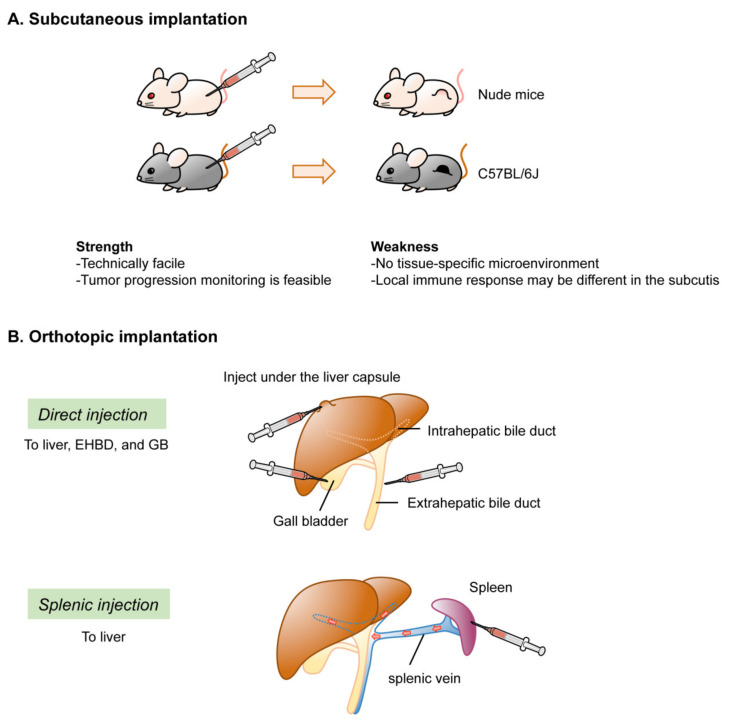
Implantation of organoids and derived tumor bud into mice. (**A**). Subcutaneous implantation. For C57BL/6J, shaving hair is not mandatory on injection, but strongly recommended upon sacrifice. (**B**). Orthotopic implantation. Only one-step injection is illustrated. Note that direct injection to EHBD might be technically feasible, but has never been reported. Splenic injection can transfer cells to the liver via splenic vein and the portal vein.

In conditional GEM models of BTC, spontaneous tumor development can occur in situ. Depending on the specificity of the Cre strain used, however, the distribution may not be limited to the target organs or cells. In contrast, subcutaneous or orthotopic implantation models can develop tumors at the inoculated site. For iCCA, methods for inoculation of the liver have been well established. However, orthotopic implantation has not yet been established for eCCA. With regard to GBC, only a few studies have reported orthotopic implantation of the GB and resultant tumor development. However, there is an opposing view of the feasibility of orthotopic tumor development by direct inoculation into the GB, and an organoid-based two-step inoculation method was recently established to model orthotopic GBC development [24]. In this subsection, we highlight the technical aspects and limitations of the implantation approach (Figure 4).

#### 2.4.1. Ectopic Implantation

Transduced organoids or cells are injected into the subcutis or kidney capsules to evaluate tumorigenicity. The advantages of using this approach in nude mice are the simplicity of the procedure and easy monitoring of tumor development. However, even if injected into syngeneic mice, aspects of the microenvironment could be considerably different between orthotopic and ectopic implantation. For example, orthotopic implantation of murine syngeneic cancer cell lines resulted in faster development of tumors that exhibited more prominent clinical symptoms, such as weight loss, cachexia, and pancreatitis, whereas subcutaneous inoculation led to the development of tumors with more prominent desmoplastic reactions and hypovascular stroma [91]. Tissue-resident macrophages, unlike monocyte-derived macrophages, are locally induced by inflammation and settle and mature in various organs from the embryonic stage [92,93]. The responses to the same cell line by tissue-resident immune cells may vary among tissues. In fact, the immunomodulatory cells that predominantly act in the subcutaneous tissues and lung metastasis are functionally distinct [94]. These differences may potentially modify the carcinogenic process and thereby critically affect drug metabolism and therapeutic efficacy. Thus, accurate evaluation of tumorigenicity in ectopic implantation models is difficult, and careful consideration is required when interpreting the results of preclinical studies.

#### 2.4.2. Orthotopic Implantation

To model iCCA, orthotopic implantation of genetically modified cells into the peripheral intrahepatic bile duct can be achieved by injection, either directly into the capsule of the liver [18,95] or indirectly into the spleen to allow cells to reach the liver via the splenic vein and engulf in the peripheral Glisson capsule. Syngeneic immunocompetent mice are usually selected as hosts for orthotopic implantation to investigate carcinogenesis in the presence of host-tumor interactions. In several studies, retrorsine and CCl4 were administered to exhaust normal hepatic stem cells and facilitate engulfment of the progenitor population to the host liver [96,97]. No reports have described orthotopic implantation models of eCCA. This might be because the extrahepatic bile ducts, such as the CBD in mouse, are too short and thin to inject organoids mixed with Matrigel.

Two methods have been reported for GBC models. One involves injecting transduced GB organoids under the liver capsule [21]. This may be difficult to regard as an orthotopic injection in a strict sense, although human GBC often invades the liver during progression. The other method involves injecting human [98,99] or mouse cells [23] directly into the lumen of the GB. However, this procedure can readily allow cells to pass through the bile duct to the Vater papilla into the duodenum. If cells are resuspended in a liquid and injected locally, there is always a risk of leakage from the injection site to the peritoneal cavity, which makes it difficult to distinguish from metastatic disease. To address these issues, a novel two-step model was recently documented for GBC. Transduced organoid-derived subcutaneous tumors in syngeneic mice were subjected to orthotopic implantation, as fragmented “tumor bud.” This approach, termed implantation of organoid-derived tumor buds (IoTB), enabled the establishment of a fine GBC model without injection-associated cell leakage [24].

## 3. In Vivo GEM Model of BTC

Owing to *Alb* expression in hepatoblasts, which are bi-potential progenitors for hepatocytes and cholangiocytes, liver-specific tumor models have been generated mostly by intercrossing *Alb*-Cre mice with conditional GEMs carrying floxed alleles. However, the histology of the resulting tumors varies among HCC, iCCA, and the mixed type, depending on the reconstitution of genetic alterations. For example, in mice with liver-specific homozygous deletion of *Smad4* and *Pten* (hereafter *Alb*-Cre; *Smad4^f/f^*; *Pten^f/f^*), all mice developed iCCA and died after 10 months, while *Alb*-Cre; *Pten^f/f^* mice predominantly developed HCC [100]. In *Alb*-Cre; *Pten^f/f^*; *Kras^LSL-G12D/+^* mice, only iCCA was observed, while both iCCA and HCC were observed in *Alb*-Cre; *Pten^f/+^*; *Kras^LSL-G12D/+^* mice [101]. Considering that many factors can affect the development of iCCA and HCC, it is challenging to generate genuine iCCA or HCC separately by genetic aberrations in a deterministic way. Other mouse models for iCCA were mixed with HCC to varying extents, including *Alb*-Cre, *Kras^LSL-G12D/+^*, *Trp53^f/f^*, *Alb*-Cre, *Kras^LSL-G12D/+^*, *Pten^f/f^*, *Alb*-Cre; *Kras^LSL-G12D/+^*; *Idh1^f/f^*, *Alb*-Cre; *Notch1*; *Kras^LSL-G12D/+^*, *Alb*-Cre; *Kras^LSL-G12D/+^*; *Fbxw7^f/f^*, and *Alb*-Cre; *Cdh1^f/f^*; *Sav1^f/f^*; *Hspd1^f/f^* [101,102,103,104,105,106,107,108,109].

*Krt19-Cre* is often used to induce genetic alterations in the biliary system, although *Krt19* expression can also be detected in many glandular epithelia and non-keratinized squamous basal cells throughout the body [110]. In contrast, *Krt19*-Cre, *Kras^LSL-G12D/+^*, *Tgfbr2^f/f^*, and *Cdh1^f/f^* mice invariably developed eCCA and *Krt19*-Cre, *Kras^LSL-G12D/+^*, and *Tgfbr2^f/f^* mice died from respiratory cancers without eCCA development [22]. These results suggest that the lack of tissue specificity could impair the relevance of BTC models, especially when other organs become more cancer-prone. There are no known genes specifically expressed in GB. Nonetheless, *Pdx1*-Cre, often used to induce pancreatic carcinogenesis, also developed GBC by crossing with *Kras^LSL-G12D/+^* mice [111]. Transgenic mice expressing rat *ErbB-2* under the *bovine keratin 5* promoter, which was aimed at developing skin tumors, also developed GBC [112,113], underscoring the oncogenic potential of *ErbB-2*.

## 4. Implantation-Based Hybrid Mouse Model of BTC

Although GEM is currently the gold standard in modeling tumorigenesis, it is still impossible to perfectly phenocopy all aspects of sporadic carcinogenesis. For example, currently available Cre mice may not provide sufficient tissue specificity, and the resultant gene recombination is homogenously observed across the target organ during development. In contrast, the tumor originates from a single mutated cell in adults, among many genetically intact normal cells in the organ. To address these issues, novel hybrid models have been developed by combining the in vitro genetic engineering of primary cells and subsequent implantation into host mice in various types of cancers, including BTC, intestine, lung, and pancreas [19,58,59,88,114,115]. This hybrid approach can also integrate GEM as a source of primary cells, thereby accelerating the modeling of BTC without multiple intercrossings to generate many GEMs. The implementation- and organoid-based BTC models are summarized in Table 1.

### 4.1. Fetal Liver-Based Model of CCA and HCC

In the fetus, the liver provides a niche for hematopoiesis [116,117]. Hematopoietic stem cells isolated from the Eμ-*myc* mouse lymphoma model were transduced with a retrovirus encoding oncogenes and transplanted into sublethally irradiated mice. Modified stem cells engulfed the bone marrow of the recipient mice, reconstituted hematopoiesis, and later developed lymphoma of various genotypes [118]. Similar to this hybrid lymphoma model, the liver version was established using the same fetal liver. Specifically, hepatic progenitor cells, which are rare in adults but abundant in the fetus (E12.5-15), were captured using E-cadherin antibody and subjected to retroviral transduction with specific genes or libraries [96,97,119]. By introducing *c-Myc*, *H-Ras*^V12^, and *myr-Akt* into *Trp53^−/−^* primary hepatoblast cells, liver tumors were generated with various histological features resembling HCC, iCCA, and hepatoblastoma [96]. Alternatively, hepatoblasts transduced with *Hras*^V12^ and Tet-off *Trp53* shRNA developed HCC or CCA in the absence of doxycycline owing to activated *Hras* and *p53* inactivation [119]. After restoration of p53 by the addition of doxycycline, the tumors rapidly regressed, verifying the huge therapeutic impact of p53 reactivation in vivo in the tumors. Hepatoblasts isolated from *Alb*-Cre; *Trp53*^LSL-R172H/f^ mice were transduced with retroviral *c-Myc* and *Kras^G12D^*, which developed HCC and CCA, respectively. CCA development from *Alb*-Cre, *Kras*^LSL-G12D/+^, and *Trp53*^LSL-R172H/f^ hepatoblasts was augmented by *PTEN* knockdown or overexpression of the *FIG-ROS* gene. Inducible depletion of the *FIG-ROS* fusion gene resulted in potent inhibition of tumor growth, validating ROS as a therapeutic target [95].

### 4.2. Organoid-Based Models of iCCA and eCCA

The application of organoid culture technology to the carcinogenesis model was first illustrated using murine intestinal cells [58]. Lentiviral knockdown of *Apc* with shRNA allowed intestinal organoids from WT mice to develop solid tumors in the subcutis of nude mice, similar to adenoma formation in *Apc*-mutant mice. Further knockdown of *PTEN* and *Trp53* resulted in tumor progression. Cre-mediated induction of *Kras^G12D^* and/or knockdown of *Apc* in intestinal organoids from *Kras^LSL-G12D/+^* mice demonstrated significant synergy between the activation of the Ras and Wnt pathways in intestinal tumorigenicity. In contrast, *Kras^G12D^* alone only temporarily developed hyperplastic aberrant crypt foci (ACF) distinct from precancerous dysplastic ACF [120]. Tumorigenicity as evaluated by the incidence, volume, and histology of the tumor was concordant with earlier studies with *Apc*-mutant GEM, mirroring the multi-step colon carcinogenesis in humans without ever generating GEM [121]. These findings raised the possibility that this approach, a combination of organoid culture, lentiviral gene transduction, and implantation in nude mice, could be generally applicable to other organs and driver mutations as well.

Indeed, adult liver-derived organoids gave rise to *Kras^G12D^*-driven subcutaneous tumors in nude mice by additional lentivirus-based knockdown of TSGs such as *Trp53*, *Apc*, *Cdkn2a*, or *Pten.* These tumors histologically range from cysts, precancerous lesions, including BilIN, and intraductal papillary neoplasm of the bile duct to advanced adenocarcinomas with desmoplasia [19]. Interestingly, the tumors were exclusively diagnosed as adenocarcinoma or its precancerous lesions without any components of HCC, in sharp contrast to the in vivo GEM for *Kras-*driven liver cancer, which mostly comprises both HCC and CCA. Peribiliary gland-derived organoids from *Kras^LSL-G12D/+^*, *Tgfbr2^f/f^*, and *Cdh1^f/f^* mice infected with lentiviral Cre also developed subcutaneous tumors resembling CCA in the subcutis of syngeneic mice, consistent with the in vivo model of the same genotype [22]. These results suggest that a liver-specific microenvironment might be dispensable, at least for *Kras-*driven CCA development. For liver organoids expressing *Pik3ca^H1047R^*, only benign cysts were induced, even if the deletion of *Trp53* or knockdown of *Cdkn2a* or *Pten* was combined, suggesting modest tumorigenicity of *Pik3ca^H1047R^* for CCA [19].

Oncogenic mutant *Ras*-driven CCA development in the liver was also obtained by orthotopic implantation of transduced liver organoids in the *Trp53^−^*^/^*^−^* [18] or *Cdkn2a^−^*^/^*^−^* [21] background, in syngeneic immunocompetent mice. Notably, the tumors retained plasticity to induce a broader differentiation spectrum of liver cancers, although organoids predominantly displayed cystic morphology and bile duct lineage differentiation. For example, overexpression of *c-myc* in liver organoids with *Trp53* knockdown and *Apc* knockout developed HCC-like tumors in the liver [18], underscoring the interplay between genetic alterations and the hepatic microenvironment. The *FGFR2*-fusion genes, frequently observed in iCCA but not in eCCA [122], could drive tumorigenesis by combining with *Cdkn2a* knockdown [19] in the subcutis of nude mice, but only with incomplete penetrance. In contrast, overexpression of various *FGFR2*-fusion genes in the *Trp53*-null liver organoids developed tumors in the liver with complete penetrance [20], suggesting a preference for the intrahepatic niche by the FGFR2 signaling pathway for tumorigenesis. Notably, specific inhibitors of FGFR2 verified that the tumor-derived organoids were indeed reliant on the pathway, providing a rationale for the molecular targeted therapy against *FGFR2*-driven iCCA.

### 4.3. Organoid-Based Model of GBC

One of the technical challenges of modeling GBC using the GEM approach is the lack of adequate Cre mice to achieve GB-specific gene recombination. To address this issue, adult murine GBs were physically isolated from *Kras^LSL-G12D/+^* mice and propagated as organoids. After lentiviral transduction with Cre, the resultant *Kras^G12D/+^* organoids did not develop any tumors in nude mice, but further knockdown of *Cdkn2a* or *Pten* resulted in the development of subcutaneous tumors resembling well-differentiated GBC, albeit with incomplete penetrance. More aggressive tumors developed by implantation of tumor-derived organoids. By deletion of *Trp53* in *Kras^G12D/+^* organoids, subcutaneous tumors resembling moderately differentiated GBC developed with complete penetrance [19]. These observations suggest that genetic cooperation critically determines the tumorigenicity of GB organoids, while it may also be modified by interactions with the tissue stroma. With regard to *Pik3ca^H1047R^* GB organoids, benign cysts were induced only if the deletion of *Trp53* or knockdown of *Cdkn2a* or *Pten* was combined [19], suggesting weaker tumorigenicity of *Pik3ca^H1047R^* than *Kras^G12D^* for GBC. Tumor development was also observed by intrahepatic injection of *Cdkn2a*^−/−^ GB organoids overexpressing mutant *KRAS* [21], although implantation to the GB was not investigated in this study. Orthotopic GBC development was documented by directly injecting the *Trp53*^−/−^ GB organoids overexpressing mutant *ERBB2* or *Kras^G12D^* into the lumen of the GB of immunodeficient mice and syngeneic mice, respectively, whereas WT *ERBB2* did not give rise to tumors [23], uncovering the oncogenic potential specific to mutations in *ERBB2*.

**Figure 5:**
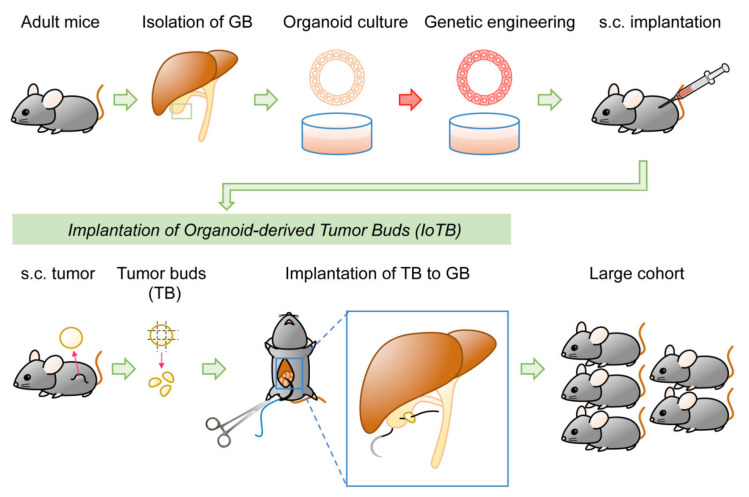
The two-step implantation model of GBC. Genetically engineered organoids were inoculated into the subcutis of syngeneic mice. In the second step, the IoTB approach facilitated accurate preclinical studies in a large cohort.

Orthotopic implantation in the abdomen can often result in leakage from the injected site, which might hamper tumor development or lead to the development of disseminated nodules in the peritoneum. In addition, digestive enzymes in the bile can degrade Matrigel, allowing organoids to flow into the CBD. To address these issues, a two-step approach is adopted. Specifically, subcutaneous tumors were first generated in syngeneic mice by implanting *Kras^G12D^*-expressing GB organoids that were subjected to CRISPR/Cas9-based deletion of *Trp53*, *p19^Arf^*, and *Smad4*. Subsequently, the minced fragments of derived tumors, called tumor buds (TBs), were sutured on the outside of the GB [24]. Orthotopic tumors highly resemble human GBC in terms of macroscopic and microscopic features and transcriptional profiles. Moreover, detailed characterization of tumor-infiltrating immune cells revealed similar profiles of immune cells in both ectopic and orthotopic tumors (Figure 5). This two-step IoTB implantation approach facilitated preclinical studies in a scalable manner, compared to the generation of many GEM mice of the same genotype by intercrossing. From the pool of three–four subcutaneous tumors, 20 TBs were generated, eventually leading to a cohort of 20 tumor-bearing mice. Notably, mice were open-inspected for accurate evaluation of the tumor volume and divided into two subgroups of equal tumor burden for treatment and non-treatment with gemcitabine [24].

## 5. Concluding Remarks

**Figure 6:**
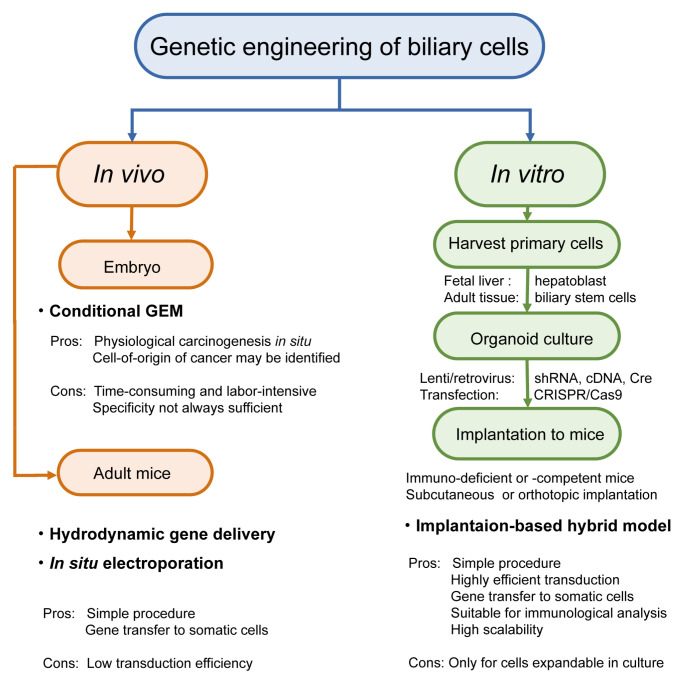
Flow chart for selection of the BTC model. Depending on the purpose of study and affordable efforts, researchers can select the most suitable experimental system for BTC modeling.

The development of highly specific Cre mice has long been sought to improve the accuracy of BTC models. By integrating organoid culture of GEM-derived cells, lentivirus-based in vitro genetic engineering, and subsequent implantation to host mice, novel BTC models have been developed. With its simple and quick nature, the generation of genetic models for BTC will be accelerated by this approach. Moreover, the recently described two-step implantation modeling further facilitated a large-scale preclinical study and could potentially pave the way to establishing orthotopic iCCA, pCCA, and eCCA models in the future. Other genetic engineering technologies can be further integrated, which will contribute to the expansion of these novel models in the BTC research field. Based on this review, researchers will be able to select the model to be used in their studies with the aid of a flow chart (Figure 6). We hope that this review will help in the development of novel innovative models based on these technologies.

## Figures and Tables

**Table 1:** Implantation- and organoid-based hybrid model of biliary tract cancer in mice.

Driver Oncogenes	Genotype of Organoids *	Methods for Genetic Engineering **		Host	Implantation	Ref.
		Oncogenes	TSGs			
**HCC (Hepatocellular Carcinoma) from Liver Organoid**						
*cMyc*	WT	cDNA (R)	shRNA (R); *Trp53* and CRISPR/Cas9 (T); *Apc*	C57BL/6J	liver	[18]
**iCCA (Intrahepatic Cholangiocelular Carcinoma) from Liver Organoid**						
*Kras^G12D^*	*Kras^LSL-G12D/+^*	Cre (L)	shRNA (L): *Cdkn2a* and/or *Pten, Trp53, Apc*	Nude	s.c.	[19]
	*Kras^LSL-G12D/+^; Trp53^flox/flox^*		N.T.			
*Pik3ca^H1047R^*	*Pik3ca^H1047R^*		shRNA (L): *Cdkn2a, Pten,*			
	*Rosa26- Pik3ca^H1047R^; Trp53^flox/flox^*		N.T.			
*FGFR2-* *AHCYL1*	WT	cDNA(R)	shRNA (L): *Cdkn2a* and/or *Pten*			
*Kras^G12D^*	*Kras^LSL-G12D/+^; Trp53^flox/flox^* (outbred)	Cre (R)	N.T. or shRNA (R): *Pten*	NSG	s.c. or liver	[18]
	*Kras^LSL-G12D/+^*	Cre (T)	CRISPR/Cas9 (T): *Pten* and *Trp53*	C57BL/6J	liver	
*FGFR2-BICC1, -MGEA5, -TACC3*	*Trp53^−/−^*	cDNA (R)	N.T.	NOD-SCID	s.c. or liver	[20]
*KRAS^G12V^*	*Cdkn2a^−/−^*	cDNA (R)		C57BL/6J	s.c., liver, or kidney	[21]
**eCCA (Extrahepatic Cholangiocelular Carcinoma) from CBD Organoid**						
*Kras^G12D^*	*Kras^LSL-G12D^; Tgfbr2^flox/flox^; Cdh1^flox/flox^*	Cre (L)	N.T.	Nude or C57BL/6J	s.c.	[22]
*KRAS^G12V^*	*Cdkn2a^−/−^*	cDNA (R)	N.T.	C57BL/6J	s.c., liver, or kidney	[21]
**GBC (Gallbladder Carcinoma) from GB Organoid**						
*Kras^G12D^*	*Kras^LSL-G12D^*	Cre (L)	shRNA (L): *Cdkn2a, Pten*	Nude	s.c.	[19]
	*Kras^LSL-G12D/+^; Trp53^flox/flox^*		N.T.			
*KRAS^G12V^*	*Cdkn2a^−/−^*	cDNA (R)	N.T.	C57BL/6J	s.c., liver, or kidney	[21]
*Kras^G12D^*	WT	Cre (T)	CRISPR/Cas9 (T): *Pten* and *Trp53*	C57BL/6J	s.c. or GB	[23]
*ERBB2^S310F or V777L^*		cDNA (R)	CRISPR/Cas9 (T): *Trp53*	NSG	s.c.	
	*Rosa26- Pik3ca^H1047R^; Trp53^flox/flox^*	Cre (L)	N.T.	Nude	s.c.	[24]
*Kras^G12D^*	*Kras^LSL-G12D^*		CRISPR/Cas9 (T): *Trp53, p19^Arf^, Smad4*	C57BL/6J	GB via s.c.	

For those models that developed cancer, pre-cancerous lesion and cystic lesions were listed. Note that liver organoid-derived tumor are either HCC or CCA (adenocarcinoma), but not mixed type liver cancer * C57BL/6J background unless otherwise indicated. ** L, lentivirus; R, retrovirus; T, transfection; N.T., not tested. Abbreviations: TSG, tumor suppressor genes; s.c., subcutis; WT, wildtype; CBD, common bile duct; GB, gallbladder.
